# Tobacco exposure inhibits SPLUNC1-dependent antimicrobial activity

**DOI:** 10.1186/s12931-019-1066-2

**Published:** 2019-05-21

**Authors:** Patrick J. Moore, Juliana Sesma, Neil E. Alexis, Robert Tarran

**Affiliations:** 1 Marsico Lung Institute, University of North Carolina at Chapel Hill, 7118A Marsico Hall, 125 Mason Farm Road, Chapel Hill, NC 27599 USA; 2 CONICET, Facultad de Ciencias Médicas, Universidad Nacional de Rosario, Rosario, Argentina; 3 Center for Environmental Medicine, Asthma and Lung Biology, The University of North Carolina at Chapel Hill, Chapel Hill, NC 27599 USA; 4 Department of Cell Biology & Physiology, The University of North Carolina at Chapel Hill, Chapel Hill, NC 27599 USA

**Keywords:** Sputum, COPD, Little cigars, BPIFA1

## Abstract

**Background:**

Tobacco smoke exposure impairs the lung’s innate immune response, leading to an increased risk of chronic infections. SPLUNC1 is a secreted, multifunctional innate defense protein that has antimicrobial activity against Gram negative organisms. We hypothesize that tobacco smoke-induced SPLUNC1 dysfunction contributes to the observed defect in innate immunity in tobacco smokers and that this dysfunction can be used as a potential biomarker of harm.

**Methods:**

We collected sputum from never-smokers and otherwise healthy smokers. We performed Western blotting to determine SPLUNC1 levels and determined antimicrobial activity against nontypeable *Haemophilus influenzae*. An in vitro exposure model was utilized to measure the effect of tobacco exposure on human bronchial epithelial culture (HBEC) antimicrobial activity against *H. influenzae*. The direct effects of cigarette and little cigar smoke exposure on SPLUNC1 function was determined using 24 h growth measurements and LPS binding assays.

**Results:**

*H. influenzae* growth in cigarette smoker’s sputum was significantly greater compared to never-smokers sputum over 24 h. HBEC supernatants and lysates contained significantly higher numbers of *H. influenzae* following chronic cigarette and little cigar smoke exposure compared to air-exposed controls. Furthermore, SPLUNC1’s antimicrobial activity and LPS-binding capability against both *H. influenzae* and *P. aeruginosa* was attenuated following cigarette and little cigar exposure.

**Conclusions:**

These data suggest that cigarette and little cigar exposure impairs SPLUNC1’s antimicrobial ability and that this inhibition may serve as a novel biomarker of harm that can be used to assess the toxicity of commercial tobacco products.

## Background

Tobacco smoke exposure is a major risk factor for several diseases including chronic obstructive pulmonary disease (COPD) [1] with 251 million cases occurring world-wide [2]. Tobacco products include cigarettes, cigars, and little cigars. A cigarette consists of a blend of tobacco encased in paper with a defined composition specification. In the US, federal regulations have defined cigars as “any roll of tobacco wrapped in tobacco leaf or any substance containing tobacco” [3]. Cigars that weigh less than 3 lbs. per 1000 units are identified as “little cigars”. Importantly, despite being of similar physical appearance as cigarettes, little cigars have evaded many of the regulations made for cigarettes. For example, whilst flavored cigarettes have been banned, flavored little cigars are still commercially available. Further, little cigars have been perceived as a safer option to cigarette smoke [4]. However, whilst the effects of little cigars in vivo have not been studied, recent data suggests that little cigars have a deleterious effect on airway epithelia that is similar or worse than cigarettes [5].

Tobacco smoke is a major risk factor for mucosal infections including pneumonia, otitis media and periodontitis. Airway surface liquid (ASL) lines the lung’s mucosal surface and includes a periciliary liquid layer and an overlying mucus layer. ASL contains approximately 1000 proteins and peptides that play a variety of roles including chemical detoxification, protection against oxidative stress, proteolysis and anti-microbial activities. In vitro, we have previously shown that chronic little cigar exposure significantly alters the ASL proteome including changes in proteins involved in the detoxification of xenobiotics and proteins required to metabolize reaction oxygen species [5]. Similarly, in vivo exposure to cigarettes leads to chronic changes to the ASL proteome [6]. These changes may be due to inflammation-induced changes in proteins expression and or altered proteolysis. These alterations are predicted to (i) impair bacterial clearance from the lung and (ii) promote colonization of the lower respiratory tract. Indeed, adult smokers are susceptible common pathogens such as *Haemophilus influenzae, Pseudomonas aeruginosa and Streptococcus pneumoniae* [7]. COPD lungs are characterized by mucus dehydration, inflammation and subsequent bacterial infections that contribute to the progressive and irreversible airway obstruction and accompanying decline in lung function [8, 9]. Consistent with their having an innate defense defect, 60% of COPD patients are chronically colonized with bacteria including *H. influenzae* [10].

Short palate lung and nasal epithelial clone 1 (SPLUNC1; gene name *BPIFA1*) is a 25 kDa protein that is secreted into the ASL by the underlying epithelia. A key component of the innate immune response to infections, SPLUNC1 has antimicrobial activity against many Gram-negative bacteria including *H. influenzae, P. aeruginosa* and the *Burkholderia cepacia* family [11, 12]. Secreted SPLUNC1 levels are diminished in chronic inflammatory diseases including cystic fibrosis and asthma [13, 14]. Additionally, SPLUNC1 may also be degraded in COPD airways by neutrophil elastase, which may predispose COPD patients to *H. influenzae* colonization, and indeed, SPLUNC1 may be a key innate defense protein against COPD pathogens [15]. Recently, we demonstrated that cigarette smoke promoted adduct binding to SPLUNC1, resulting in a loss of its ability to regulate ASL hydration [16]. These data suggested that there may be a “double hit” where SPLUNC1 is inactivated by smoke exposure adducts and/or degraded by neutrophil elastase. However, the impact of tobacco smoke on SPLUNC1’s antimicrobial activity against COPD-relevant pathogens has yet to be determined. Here, we therefore tested the effect of cigarette and little cigar exposure on SPLUNC1’s antimicrobial abilities.

## Methods

### Collection of Normal and smoker sputum samples

Induced sputum samples were collected as per the UNC IRB protocol #13–3454. Sputum samples were obtained as described previously [14]. In brief, subjects inhaled 3, 4 and 5% hypertonic saline, each for a 7 min period. To reduce squamous cell contamination, all subjects performed a 3-step cleansing procedure, including rinsing and gargling of the mouth with water, clearing of the throat without coughing, and blowing of their nose. Following cleansing, induced sputum samples were collected into specimen cups using a cough from the chest. Samples were incubated in Dulbecco’s Phosphate Buffered Saline solution with agitation for 15 min followed by centrifugation and collection of supernatant. The demographics and cytology pertaining to the subjects selected for this study are shown in Table 1.

**Table 1:** Demographics and cytological characteristics of sputum for the subjects used in this study

Sputum Samples	Never-Smokers	Smokers
Male/Female	5/1	4/2
Ethinicity (Hispanic/Non- Hispanic)	5/1	0/6
Age at bronch	31 ± 5.19	29 ± 6.9
Pack Years	0.00 ± 0.00	8.03 ± 6.82
BMI	27.86 ± 7.22	23.36 ± 3.57
FVC%	92.5 ± 7.23	101.6 ± 12.1
FEV_1_	95 ± 8.29	92 ± 11.47
%polymorphonuclear cells	39 ± 9.07	77.9 ± 10.20**
%macrophages	59.5 ± 8.95	20.2 ± 8.90**
%eosinophils	0.35 ± 0.41	0.68 ± 0.95
%lymphocytes	0.00 ± 0.00	0.00 ± 0.00
%bronchial epithelial cells	1.11 ± 1.37	1.20 ± 1.70
%squamous cells	9.22 ± 8.19	28.87 ± 21.43

Data presented are mean ± SD. ** denotes *P <* 0.001 different to never-smokers (Mann-Whitney U-test)

### Determination of SPLUNC1 and neutrophil elastase in sputum samples

Neat sputum samples were denatured in the presence of 2.5% β-mercaptoethanol at 95 °C for ~ 10 min and were subjected to Western blotting. In brief, samples were transferred to PDVF membranes and blocked using 5% skimmed milk in Tris-buffered saline with Tween 20 (TBST-T). For detection of neutrophil elastase, membranes were probed using a mouse-monoclonal anti-hELA2 antibody, raised against residues M1 - N252 (1:3000, R&D systems), primary antibodies were detected using an anti-mouse horseradish peroxidase (HRP) conjugated secondary antibody (Thermo-Fisher Scientific). Membranes were then stripped, re-blocked and re-probed for SPLUNC1 using a goat polyclonal hPLUNC1 antibody raised against residues Q20 - V256 of hPLUNC1 (1:3000, R&D systems), a secondary anti-goat HRP (Thermo-Fisher Scientific) conjugated antibody was used for detection of hPLUNC1. Secondary antibodies were detected by enhanced chemiluminescence (Thermo-Fisher Scientific).

### Human bronchial epithelial cells (HBECs)

Cells obtained from otherwise healthy individuals were harvested via enzymatic digestion in the presence of antibiotics from human lungs deemed unsuitable for transplantation as per the UNC protocol #03–1396 [17]. Freshly isolated HBECs were seeded on 12 mm culture inserts (12 well hanging inserts, 0.4 μm pore; Corning, USA) and were maintained at the air-liquid interface for 4 weeks in a modified bronchial epithelial growth medium at 37 °C/5% CO_2_ in a humidified incubator. Donor demographics are shown in shown in Table 2.

**Table 2:** Demographics of never-smoker HBEC donors used in this study

Donor	Smoking History	Age	Sex	Ethiniticy
1	Never Smoker	48	Male	Caucasian
2	Never Smoker	58	Female	Caucasian
3	Never Smoker	69	Male	Caucasian

### SPLUNC1 purification

A plasmid containing SPLUNC1 cDNA was transformed into BL21-Codon Plus competent cells (Agilent Technologies) and recombinant SPLUNC1 (referred to as rSPLUNC1) was purified as previously described and stored at − 80 °C until required [18].

### Cigarette smoke exposure

All tobacco smoke was generated using a Borgwaldt LC1 smoke engine using a 1 × 35 ml puff every 30 s with a butt length of 36 mm (∼13 puffs over ∼5 min). We generated smoke from Kentucky Research Cigarettes (code 3R4F, Class A cigarettes). Commercially available Marlboro and Camel cigarettes were also studied. Since little cigars were recently found to have a markedly different chemical profile to cigarettes [5], several types of little cigars including as Swisher Sweets Original, Swisher Sweets Strawberry, Captain Black, Cheyenne and Djarum cigars were studied. For our chronic epithelial tobacco smoke exposure, HBECs were placed in a chamber that exposed the apical but not the basolateral surface to cigarette smoke. Cells were then exposed to smoke from 1 cigarette or little cigar or an air control exposure every day for 5 days and washed with PBS daily after each exposure, with daily changes of serosal media [5]. To study rSPLUNC1 under cell-free conditions, the protein was dissolved in Ringer’s solution and 100 μl of this solution was placed in a Petri dish and exposed to tobacco smoke.

### HBEC infection and antimicrobial assays

Nontypeable *H. influenzae* (referred to hereon as *H. influenzae*) was grown in brain heart infusion (BHI) broth supplemented with 1 mg/ml hemin and 10 μg/ml nicotinamide adenine dinucleotide (sBHI) at 37 °C for 24 h with shaking at 300 rpm. *P. aeruginosa* strain PAO1 was grown overnight in Luria broth (LB) at 37 °C for 24 h with shaking at 300 rpm. Colony forming units (CFU/ml) were determined by serial dilution on chocolate agar plates. The bacterial cultures were adjusted to an optical density at 600 nm (OD_600_) of ~ 0.600, and 10^6^ CFU/ml bacteria were apically added to chronically tobacco exposed HBECs for 2 h. Apical supernatants and lysates of HBECs were collected, serially diluted, plated on LB agar plates and incubated at 37 °C for 24 h to determine CFU/ml. For the antimicrobial assay, 10^6^ CFU/ml of *H. influenzae* and *P. aeruginosa* were incubated with tobacco exposed and air exposed rSPLUNC1 (10 μM) in flat clear bottom 96-well plates (Corning Incorporated) for 24 h at 37 °C. Samples were collected at 24 h, serially diluted in Ringer’s solution and then plated on LB agar plates to determine CFU/ml.

### Extraction and quantitation of *H. influenzae* lipopolysaccharide

Lipopolysaccharide (LPS) was extracted from *H. influenzae* and *P. aeruginosa* strain 10 (MilliporeSigma) using the phenol-water method as described previously [19]. Briefly, biomass from 2 chocolate agar plates was harvested into PBS after 72 h in culture. Bacteria were washed 3 times in PBS and collected by centrifugation (4000 x g for 10 min at 4 °C). The bacterial pellet was then washed once in deionized H_2_O and collected by centrifugation. Following washes, the pellet was resuspended in 750 μl of deionized H_2_O and an equal volume of 90% phenol (vol/vol; pre-heated to 65 °C; MilliporeSigma) was added. The sample was mixed for 1 min using a vortex mixer and then incubated for 10 min at 65 °C with regular mixing. Following incubation, the sample was cooled on ice and then centrifuged at 12,000 x g for 10 min at 4 °C. Extracted LPS was then treated with 200 μg of deoxyribonuclease II (Roche) and ribonuclease A (MilliporeSigma) for 30 min at 37 °C, then incubated with 200 μg proteinase K (MilliporeSigma) for 1 h at 60 °C and finally heated to 90 °C for 3 min. Extracted LPS was then quantified using the Purpald assay as previously described [20].

### LPS binding assays

A modified, enzyme-linked immunosorbent assay (ELISA)-based LPS binding method was used to detect interactions between LPS and SPLUNC1 as described previously [11]. Briefly, 96-well plates were coated overnight with purified LPS (400 ng) from *H. influenzae* and *P. aeruginosa* strain 10 (MilliporeSigma). Wells were washed and blocked with 1% bovine serum albumin (BSA)– PBS for 1 h, then, 400 ng of purified SPLUNC1 was added to each well in triplicate. PBS was used as a control for this experiment. An antibody specific to human SPLUNC1 (R&D Systems) diluted 1:5000 with BSA, was used to detect the LPS-bound SPLUNC1. Horseradish peroxidase-conjugated anti-goat antibody was used as the secondary antibody to detect binding rSPLUNC1. Enzyme activity was detected using a TMB Ultra 1-step assay (Pierce Biotechnology) and reaction was stopped with H_2_SO_4_ (Fisher). Absorbance was detected at OD_450nm_ in a BioTek spectrophotometer (BioTek).

### Statistical analysis

The number of replicates performed per experiment is noted in the respective figure legends. All experiments were repeated on ≥3 separate occasions. All experiments that were conducted using HBECs were repeated using 3 different donors on separate occasions with triplicates per donor unless otherwise indicated. Data are shown as mean ± standard error. Differences between means were tested for statistical significance using Mann-Whitney t-test, analysis of variance (ANOVA), Kruskal-Wallis test with Dunn’s multiple comparison and two-way ANOVA with Tukey’s multiple comparisons test as appropriate. Statistical analysis was performed using GraphPad Prism 7.0 with *p* < 0.05 considered as being significant.

## Results

### Smokers sputum contained altered cytological measurements

The demographics and cytology pertaining to the subjects selected for this study were summarized in Table 1. Cigarette smokers had an 8.03 ± 6.82 pack year smoking history. All smokers were healthy and there was no significant difference in FVC% and FEV_1_% between the groups. In contrast, sputum cytology analysis revealed a statistically significant difference in polymorphonuclear cells and macrophages in smokers’ sputum (Table 1).

### *H. influenzae* proliferates in cigarette-smokers sputum

**Fig. 1:**
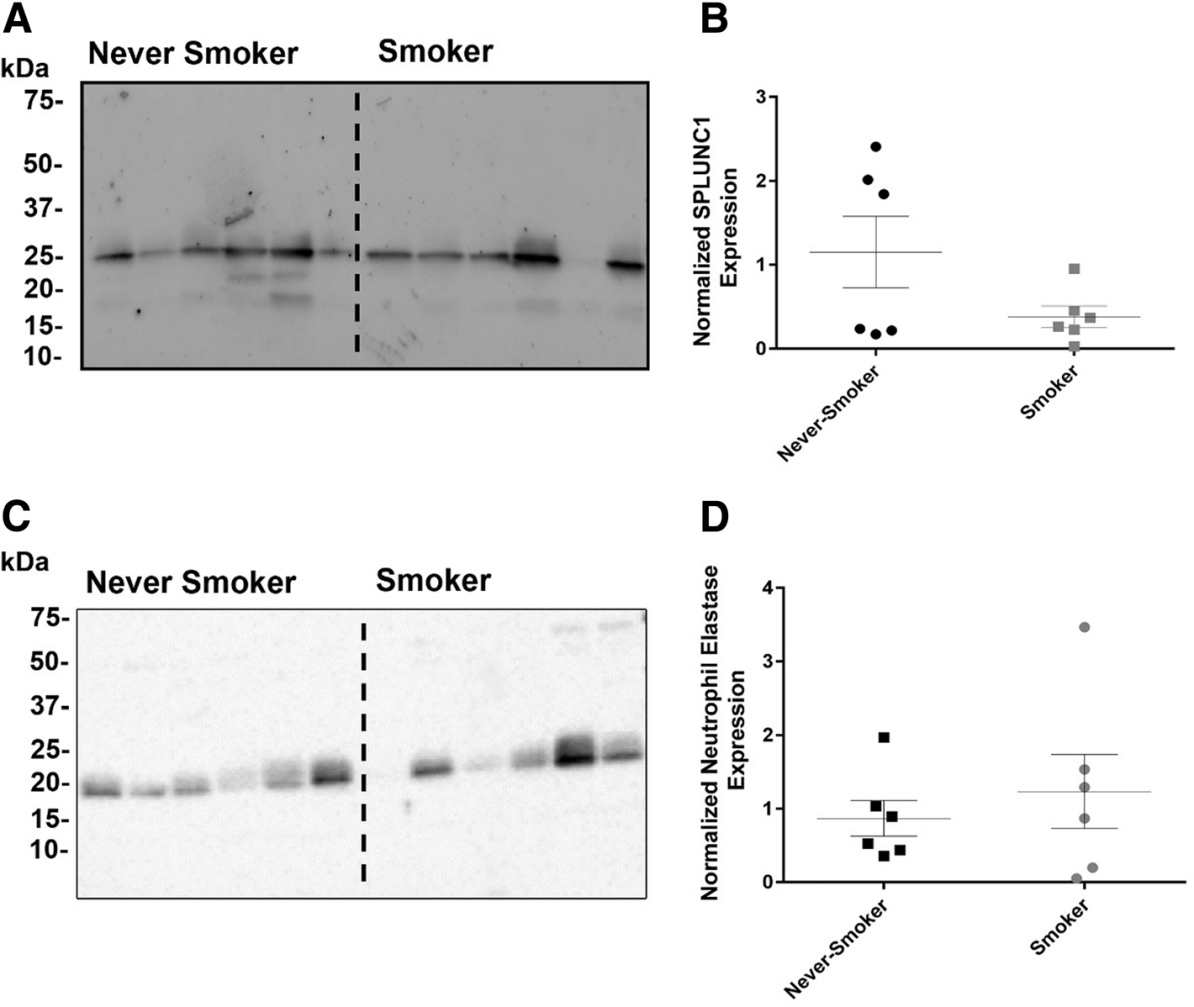
SPLUNC1 protein levels are similar in smokers’ and never-smokers’ sputum. Representative Western blots showing endogenous expression of SPLUNC1 (**a**) and neutrophil elastase (**c**) in never smoker and smokers sputum from n = 6 donors per group. Membranes were probed for SPLUNC1 prior to stripping and re-probing for neutrophil elastase. Bar graphs showing mean densitometry of SPLUNC1 (**b**) and neutrophil elastase (**d**) protein abundance in never smokers and smokers sputum

**Fig. 2:**
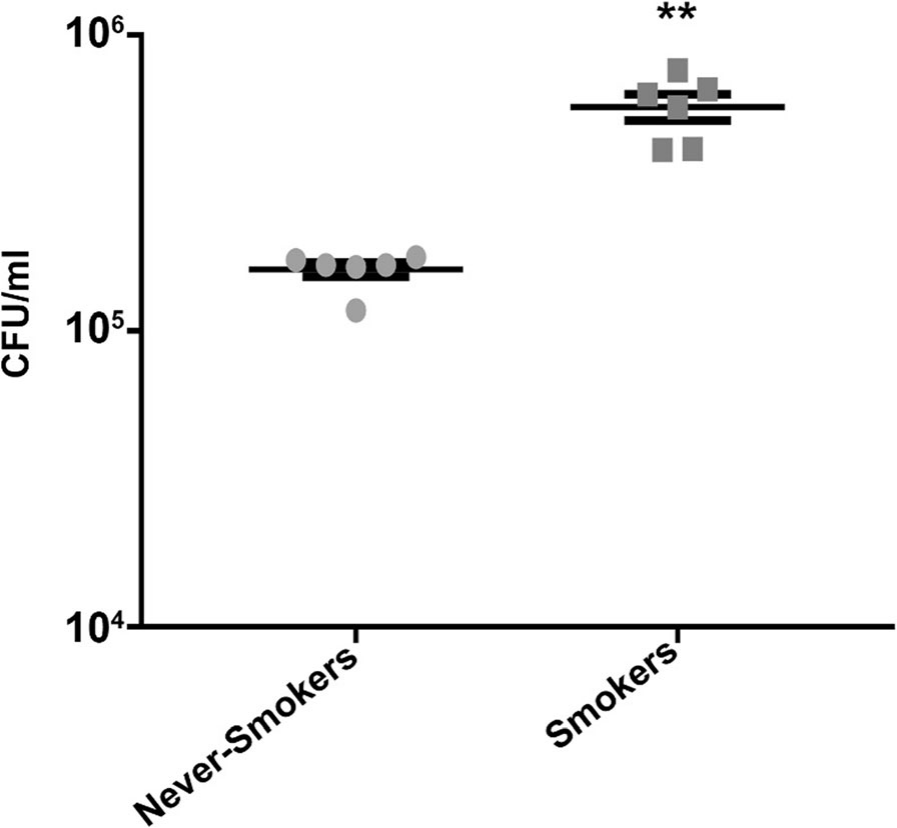
Cigarette smoker’s sputum has increased *H. influenzae* growth. Sputum was obtained from never-smokers and smokers (*n* = 6 per group). 10^5^ CFU *H. influenzae* were added to 20 μl of Sputum and CFUs were measured 24 h later. **p < 0.001 different to never-smokers (Mann-Whitney *U*-test)

We first determined whether SPLUNC1 was present in smoker’s sputum. Consistent with our goal of recruiting “healthy smokers”, we detected no significant difference in SPLUNC1 levels in never-smokers’ and smokers’ sputum (Fig. 1). Similarly, neutrophil elastase was present in both groups and was also not significantly different (Fig. 1). We next interrogated the antimicrobial activity of smokers’ vs. never-smokers’ airway secretions by culturing them with *H. influenzae* for 24 h. We found that significantly more *H. influenzae* grew in smoker’s sputum compared to never-smoker’s sputum (Fig. 2).

### Chronic cigarette and Little cigar smoke exposures cause increased *H. influenzae* growth

**Fig. 3:**
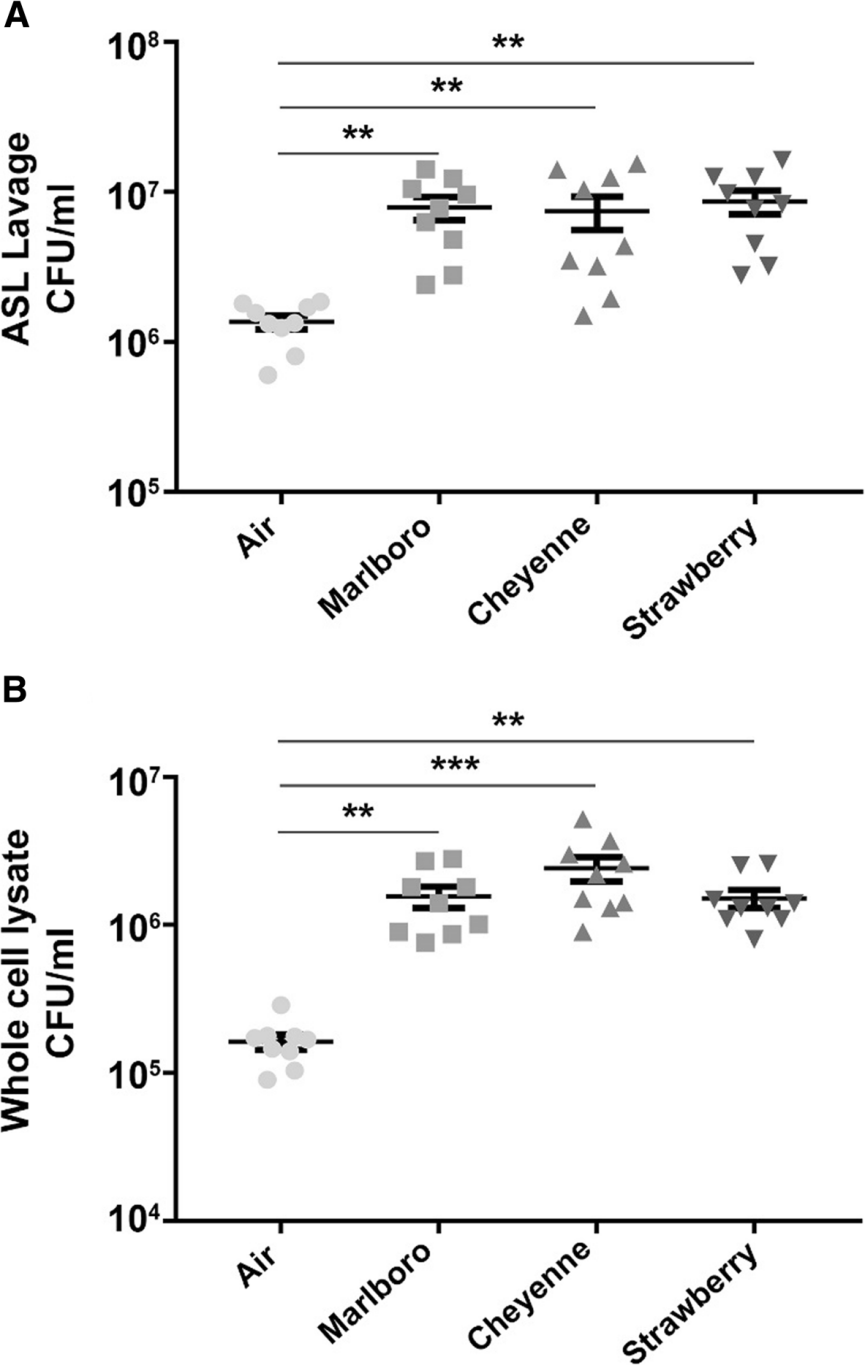
Tobacco smoke exposure leads to increased *H. influenzae* colonization. HBECs obtained from normal donors were exposed to air or tobacco smoke from Marlboro cigarettes, Cheyenne little cigars and Swisher Sweets Strawberry little cigars, once a day for 5 days. 20 μl of *H. influenzae* at 10^6^ CFU/ml were added apically to HBECs for 2 h at 37 °C/5% CO_2_. **a** Apical supernatants and (**b**) lysates were collected, serially diluted, plated on LB agar plates and incubated at 37 °C for 48 h to determine colony-forming units (CFU) for bacterial load (all *n* = 9). Statistically significant differences were measured using the Kruskal-Wallis test. **p < 0.001, ***p < 0.0001 different to air control

To assess the impact of different tobacco products on human airway epithelia, we chronically exposed HBECs to a commercial cigarette (Marlboro) and two little cigar products (Cheyenne and Swisher Strawberry) or air (control) for 5 days. We have previously observed that chronic (5 day) exposure from either cigarettes or little cigars did not cause gross cellular abnormalities [5] and consistent with this observation, all cultures remained viable throughout the exposure period. After the chronic exposure, HBECs were infected mucosally with *H. influenzae* for 2 h, then lavages and whole cell lysates were collected after incubation. Interestingly *H. influenzae* colonization of tobacco smoke-exposed HBECs was significantly increased compared to the air-exposed culture (Fig. 3a). In addition, we also recovered significantly higher numbers of internalized *H. influenzae* from lysates of tobacco-exposed cultures (Fig. 3b).

### Tobacco exposure to SPLUNC1 attenuates bacteriostatic ability

**Fig. 4:**
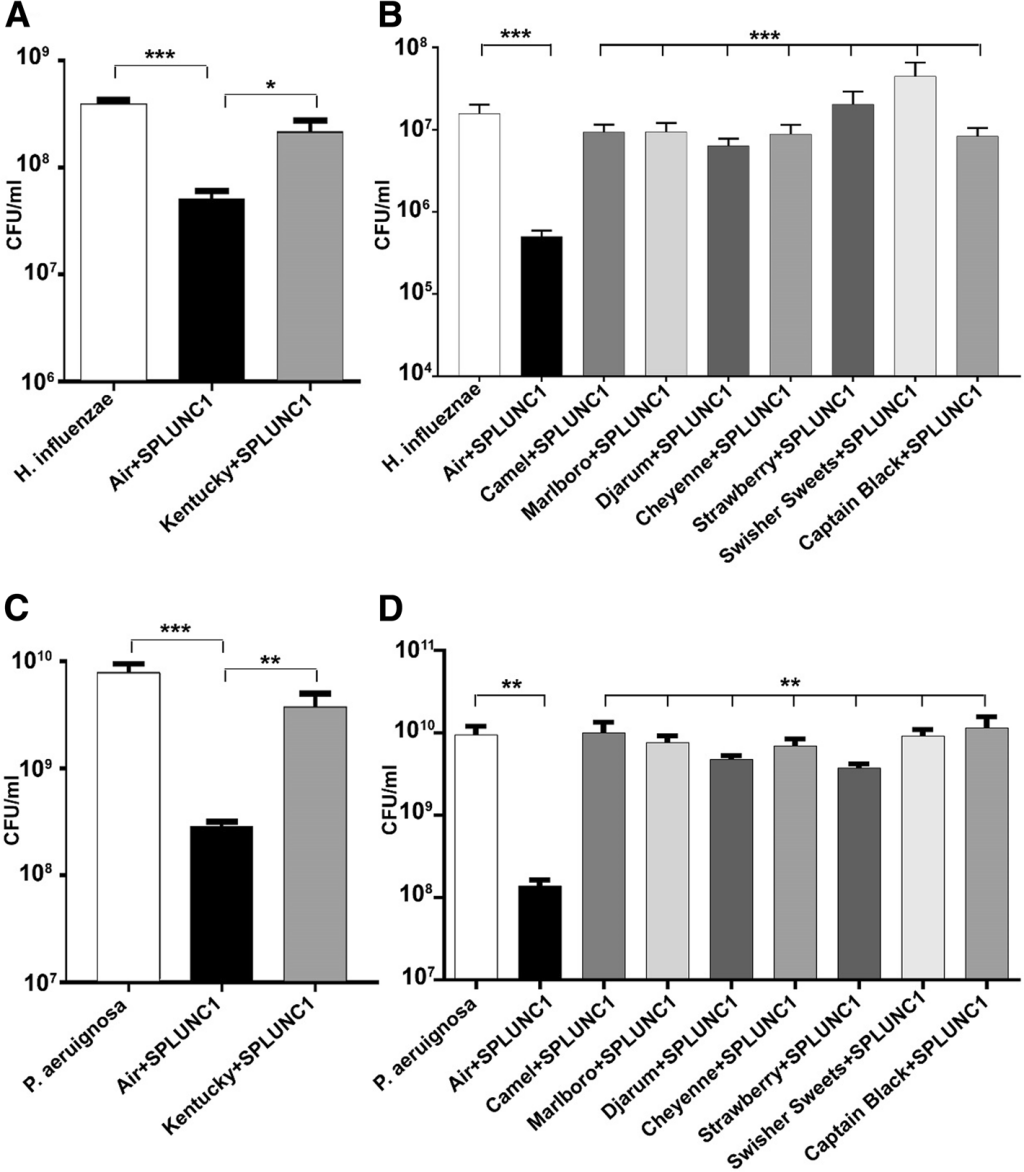
Tobacco smoke exposure reduces SPLUNC1 bacteriostatic activity. **a**, **c** 10 μM of SPLUNC1 or Kentucky cigarette-exposed SPLUNC1 dissolved in Ringer’s solution were co-incubated with 10^6^ of *H. influenzae* or *P. aeruginosa* for 24 h (all *n* = 3 per group). **b**, **d** *H. influenzae* or *P. aeruginosa* were co-incubated with 10 μM of SPLUNC1 or tobacco-exposed SPLUNC1 for 24 h; Camel, Marlboro, Djarum, Cheyenne, Swisher Sweets, Swisher Sweets Strawberry, and Captain Black (all *n =* 3 per group). Statistically significant differences were measured using Kruskal-Wallis test. *p < 0.05, **p < 0.01, ***p < 0.001 different to control

We have previously demonstrated that SPLUNC1’s ability to regulate ASL homeostasis was attenuated following exposure to cigarette smoke [16]. However, the effects of cigarette smoke on SPLUNC1’s antimicrobial activity have not yet been determined. Therefore, to test whether cigarette smoke from Kentucky research cigarettes altered SPLUNC1’s antimicrobial functions, we used a physiological concentration (10 μM) of rSPLUNC1 [21]. After exposure to air or tobacco smoke, we incubated rSPLUNC1 with *H. influenzae* for 24 h. rSPLUNC1’s antimicrobial activity was attenuated and *H. influenzae* growth was significantly greater in the presence of tobacco exposed-SPLUNC1 compared to air-exposed SPLUNC1 (Fig. 4a). To determine whether other tobacco products also attenuated SPLUNC1’s antimicrobial functions, we exposed rSPLUNC1 to commercial cigarettes including Marlboro and Camel cigarettes, as well as little cigars such as Djarum, Cheyenne, Swisher Sweets Original, Swisher Sweets Strawberry, and Captain Black. Consistent with the effects seen with Kentucky research cigarettes, commercial tobacco products also significantly attenuated SPLUNC1’s antimicrobial activity (Fig. 4b). To test whether this effect extended beyond that seen with *H. influenzae*, we also tested the ability of tobacco smoke to impair SPLUNC1’s antimicrobial actions against *P. aeruginosa* [22]. Similar to the results obtained with *H. influenzae,* SPLUNC1’s antimicrobial activity against *P. aeruginosa* was also attenuated following tobacco exposure (Fig. 4c, d), suggesting that this phenomenon may be relevant to multiple Gram-negative organisms.

### SPLUNC1 LPS-binding properties are reduced after cigarette and Little cigar smoke exposure

**Fig. 5:**
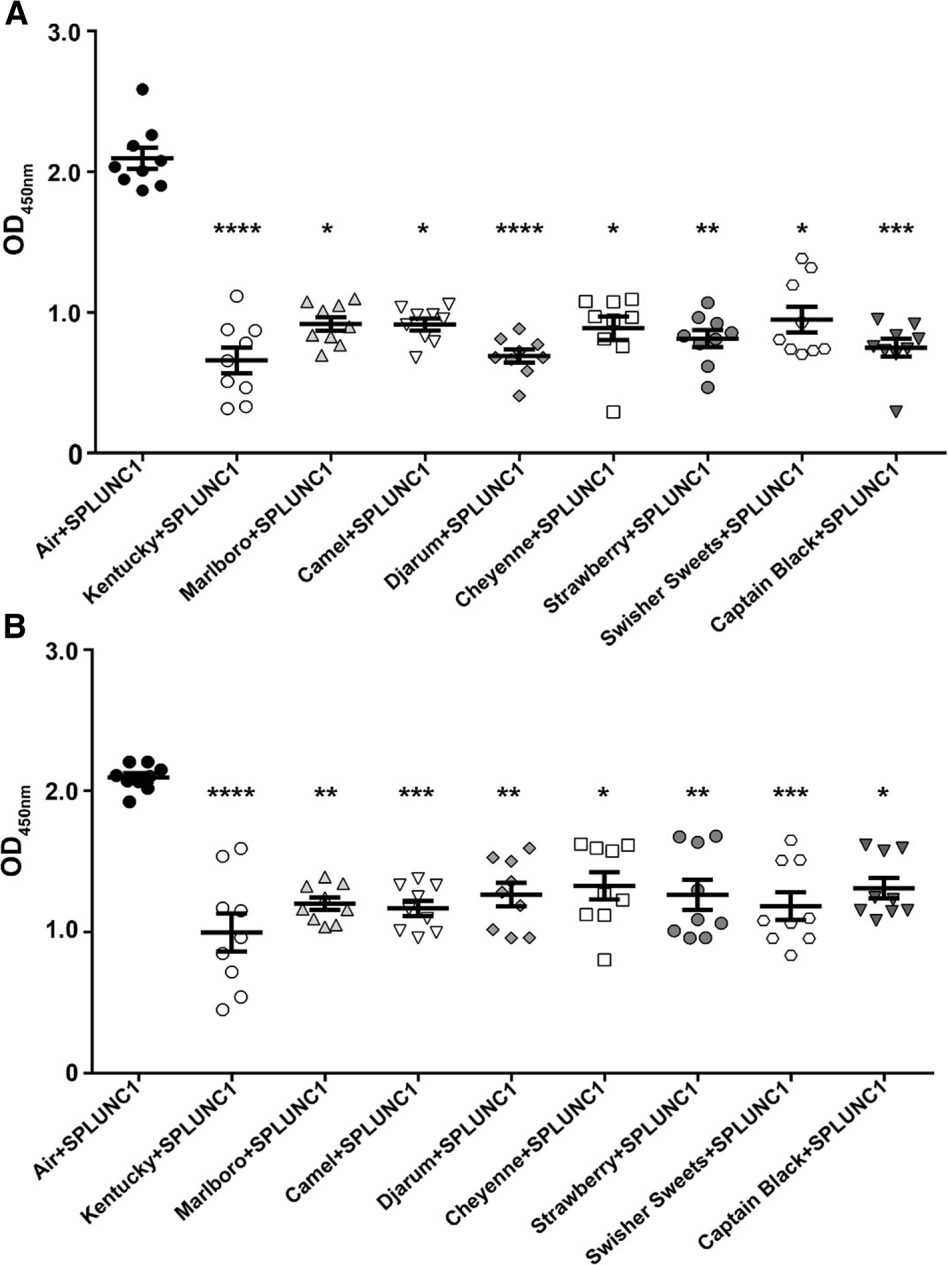
Tobacco smoke reduces SPLUNC1’s ability to bind LPS. Graphs show Non-linear regression fit generated from one site specific binding of LPS derived from either *H. influenzae* (**a**) or *P. aeruginosa* (**b**) with 2 fold dilutions of SPLUNC1, cigarette and tobacco smoke exposed SPLUNC1 dissolved in Ringer’s solution to commercial cigarettes and several little cigars (all *n* = 9 per group). Statistically significant differences were measured using Kruskal-Wallis test. *p < 0.05, **p < 0.01, ***p < 0.001, ****p < 0.0001 different to Air + SPLUNC1

SPLUNC1 has previously been shown to bind LPS from different Gram-negative bacteria, which may be linked to its antimicrobial activities [11]. However, the effect of tobacco smoke exposure on SPLUNC1-LPS interactions has not been studied. We therefore purified LPS from both *H. influenzae* and *P. aeruginosa* and determined SPLUNC1-LPS binding using an ELISA-based assay [23]. Consistent with previous observations [11], we found that SPLUNC1 bound LPS in a dose-dependent fashion (Fig. 5). Indeed, following exposure to Kentucky research cigarettes, commercial cigarettes (Camel and Marlborough) and little cigars (Djarum, Cheyenne, Swisher Sweets and Captain Black), we observed a significant reduction in SPLUNC1 binding to LPS purified from *H. influenzae and P. aeruginosa* (Fig. 5a, b).

## Discussion

ASL contains multiple anti-microbial proteins and peptides, as well as proteases and mucins, all of which help to clear inhaled bacteria, and it is likely that the system redundancy is an important feature to combat against bacterial colonization. It was recently demonstrated that 44 proteins had altered abundance in sputum from cigarette smokers compared to never-smokers, including the MUC5AC mucin and a number of antioxidant and xenobiotic metabolizing proteins [24]. These authors also detected increases in anti-microbial proteins such as lysozyme and lactotransferrin in cigarette smokers sputum [24]. Despite their being numerous proteins in the ASL, we and others postulate that SPLUNC1 is critically important for innate defense. For example, it has previously been demonstrated that SPLUNC1 knockout mice exhibited increased susceptibility to infection and failed to clear *H. influenzae* and *P. aeruginosa* from their lungs [15, 25]. Jiang et al. further speculated that SPLUNC1 plays a key role in preventing *H. influenzae* colonization of the lung, and that SPLUNC1’s activity is impaired in COPD patients [15]. Based on these data, we used SPLUNC1 as a representative anti-microbial agent and tested its sensitivity to tobacco smoke exposure. We found that both never-smokers and cigarette smokers’ sputum contained similar levels of SPLUNC1 protein (Fig. 1). Consistent with neutrophil elastase being the predominant protease that degrades SPLUNC1 in ASL [15, 26] total neutrophil elastase protein levels were also similar between never-smokers and smokers (Fig. 1). These data are similar to the Reidel study where they also found no change in SPLUNC1 and neutrophil elastase despite seeing a similar increase in sputum neutrophil levels [24] (Table 1).

Despite our observing no change in SPLUNC1 levels, and other researchers finding an increase in anti-microbial proteins in smoker’s sputum, we observed a significant increase in *H. influenzae* growth in smokers’ sputum (Fig. 2), suggesting that innate immune defense was impaired and we went on to study this phenomenon in more detail in vitro. It has recently been demonstrated that bronchoalveolar lavage (BAL) from smokers had increased *Staphylococcus aureus and P. aeruginosa* growth compared to never-smokers’ BAL Further, *S. aureus* and *P. aeruginosa* exhibited increased rates of biofilm formation in smokers’ BAL compared to never-smokers’ BAL [27]. Taken together, these data suggest that smoker’s antimicrobial defenses were impaired. Using a proteomic approach, Qu et al. found that *H. influenzae* upregulated antioxidant/stress response proteins as well as proteins involved in the uptake of minerals such as iron and zinc [28]. These metabolic adaptations reveal critical virulence factors that enable *H. influenzae* survival in oxidative and nutritionally limited environment of the COPD lung. In the current study, we utilized sputum from non-smokers who were all never-smokers and healthy current smokers and looked primarily for sensitivity to *H. influenzae*.

It has previously been postulated that *H. influenzae* colonization contributes to the pathogenesis of COPD [29]. Crucially, *H. influenzae* can alter its genome during airway colonization, leading to changes in virulence, which may facilitate its ability to adapt to the harsh environment of the lung. For example, Pettigrew et al. observed large-scale genome rearrangement between the loci for HMW1 and HMW2 adhesins, which could influence how *H. influenzae* can attach to epithelia [30]. In addition, changes in simple sequence repeats were the main driver of change during *H. influenzae* colonization of the COPD lung [30]. Chronic colonization/adaptation of *H. influenzae* in COPD airways has been well documented, although, the underlying host defects that contribute to this phenomenon are poorly understood. Conversely, the airway microbiome of “healthy smokers” is less studied. However, COPD patients have a different lower airways microbiota than both never-smokers and smokers. This suggests that this is an evolving situation and that the lower airway microbiota changes with the development of COPD and with its progression [31].

In addition to our sputum studies, we also utilized an in vitro chronic tobacco smoke exposure system where HBECs were cultured for up to 8 weeks and maintained similar properties as native airway epithelia including similar density of ciliated cells and an isotonic ASL that mimic in vivo, suggesting that their use is valid [32]. Using this model, we previously exposed the apical but not basolateral membranes of primary HBECs to Kentucky research cigarettes as well as commercial cigarettes and little cigars [5]. We found that chronic tobacco exposure significantly changed gene expression, reduced transepithelial electrical resistance, increased interleukin (IL)-8 secretion and decreased cilia length [5]. However, after chronic tobacco exposure, the cultures remained viable and capable of maintaining an (albeit diminished) ASL. Similarly, cigarette smoke condensate suppressed IL-6, IL-8 and mitogen activated protein kinase responses to *H. influenzae* in the adenocarcinoma A549 alveolar cell line [33]. However, A549 cells do not differentiate into ciliated/goblet cells and may not fully reprise the pseudostratified epithelia seen in the conducting airways. Therefore, we postulate that having a well-differentiated cell culture model is crucial in order to determine the clinical effects of chronic tobacco exposure on host cell response to bacterial colonization. To further test the hypothesis that tobacco smoke attenuates innate antimicrobial activity, we utilized this chronic exposure system. Similar to our observations using ex vivo patient samples (Figs. 1 and 2), we observed significantly greater bacterial numbers in chronically tobacco-exposed HBECs (Fig. 3a, b), suggesting that tobacco smoke impairs antimicrobial activity within the ASL. To the best of our knowledge, this is the first time that this model has been used to observe the effects of chronic tobacco exposure on *H. influenzae* colonization of HBECs.

To better the understand effect of cigarette and little cigar smoke exposures on SPLUNC1, using a cell-free approach, we exposed recombinant SPLUNC1 to cigarette smoke and tobacco exposure as previously described [34], and then tested its antimicrobial activity against *H. influenzae* and *P. aeruginosa*. Interestingly, we observed a significant loss of SPLUNC1’s antimicrobial activity after both cigarette and little cigar smoke exposures (Fig. 4a, b). However, SPLUNC1’s antimicrobial function was similarly impaired after exposure to tobacco smoke from both cigarettes and little cigars (Fig. 4). It has previously been proposed that SPLUNC1 exerts its antimicrobial effects by binding LPS and disrupting bacterial cell walls. Consistent with previous observations [11], we found that SPLUNC1 dose-dependently bound to both *H. influenzae* and *P. aeruginosa* LPS (Fig. 5). However, after cigarette smoke and little cigar exposure, SPLUNC1-LPS interactions were also significantly attenuated (Fig. 5).

In our previous study, we found that both cigarette and little cigar exposure affected gene expression. Further, using proteomics, we found that 50 secreted/ASL proteins were significantly altered by cigarette exposure, whilst 132 proteins were altered in the little cigar exposure groups [5]. Many of these proteins were involved in detoxification of reactive oxygen species, metabolism of xenobiotics, vesicle transport and cell migration/would healing, which is consistent with repeated exposure to a toxic insult [5]. However, despite the disparity in changes between cigarette and little cigar smoke exposures, we did not observe significant changes in known antimicrobial peptides/proteins and secreted SPLUNC1 levels were not different after chronic tobacco smoke exposure [5], which is similar to our ex vivo data (Fig. 1). We have previously demonstrated that reactive aldehydes present in cigarette smoke bind to SPLUNC1’s two cysteine residues, resulting in an alteration of SPLUNC1’s quaternary structure via disruption of the disulfide bonds [16]. This interaction abrogates SPLUNC1’s ability to bind to and regulate the epithelial Na^+^ channel, ENaC [16]. Although the role of these cysteine residues in SPLUNC1’s antimicrobial function is unclear, we hypothesize that disruption of the disulfide bond may play a key role in the observed loss of SPLUNC1’s bacteriostatic activity by preventing LPS-binding.

Although the hazardous effects of cigarette smoke on lung health have been well studied [35, 36], there is a lack of knowledge regarding the effects of little cigars on lung health, and in vivo data is critically lacking. Moreover, the sales of little cigars are on the rise, especially amongst young adults, where they are perceived as a safer alternative to cigarettes [37]. Further, in some states, including North Carolina, South Carolina and Georgia, little cigars are taxed at a significantly lower rate than cigarettes and are hence seen as a cheaper alternative to cigarettes [38]. However, building on our previous studies [5, 39], our data demonstrate that little cigars do constitute a reduced risk of tobacco exposure that equally predispose the airways to risk of bacterial infections.

## Conclusions

In conclusion, our results advance the understanding of how multiple, relevant commercial brands of tobacco impair SPLUNC1’s antimicrobial function. Due to the importance of bacterial clearance for lung health, our data suggest that the loss of antimicrobial function after tobacco exposure may predispose smokers to infections and that little cigar use may similarly put smokers at risk of chronic lung infections.

## Abbreviations

ASL: Airway surface liquid
BAL: Bronchoalveolar lavage
BAL: Bronchoalveolar lavage
BHI: Brain heart infusion
BPI: Bactericidal/permeability-increasing protein
CFU: Colony forming units
COPD: Chronic obstructive pulmonary disease
ELISA: Enzyme-linked immunosorbent assay
HBECs: Human bronchial epithelial cells
IL: Interleukin
LPS: Lipopolysaccharide
SPLUNC1: Short palate lung and nasal epithelial clone 1
